# MAPPs assays for non-clinical immunogenicity risk assessment: best practices recommended by the European immunogenicity platform

**DOI:** 10.3389/fimmu.2025.1690101

**Published:** 2025-10-10

**Authors:** A.C. Karle, K. L. Kopp, R.J. Seward, S. Tourdot, C. Ackaert, M. Gutknecht, E. Cloake, N. Smith, A. Ducret

**Affiliations:** ^1^ Immunogenicity and Mechanistic Immunology, Biomedical Research, Novartis Pharma AG, Basel, Switzerland; ^2^ Centre for Functional Assays and Screening, Research and Early Development, Novo Nordisk A/S, Måløv, Denmark; ^3^ Pharmacokinetics, Dynamics and Metabolism, Pfizer Research and Development, Pfizer Inc., Andover, MA, United States; ^4^ IQVIA Laboratories In Vitro Immunology (ImmunXperts), Gosselies, Belgium; ^5^ Bioassay Department, Abzena Ltd, Babraham Research Campus, Cambridge, United Kingdom; ^6^ Early Development Services, Lonza Biologics Plc, Cambridge, United Kingdom; ^7^ Pharmaceutical Sciences, Roche Innovation Center Basel, Basel, Switzerland

**Keywords:** MAPPs, immunogenicity, risk assessment, candidate ranking, harmonization strategy

## Abstract

The use of the MAPPs (Major histocompatibility complex Associated Peptide Proteomics) assay by pharmaceutical companies, service providers, and academic laboratories is rapidly increasing, attesting to its increasingly pivotal role in biotherapeutic drug candidate design, selection, and mechanistic investigations. Implementation of the MAPPs assay is labor-intensive, necessitating a high level of expertise. Differences observed in protocols established by laboratories may lead to considerable variability in data quality, limiting inter-laboratory comparisons. To address these challenges, the Non-Clinical Immunogenicity Risk Assessment working group (NCIRA) of the European Immunogenicity Platform (EIP) sought to provide comprehensive recommendations for establishing robust workflows that will ensure robust data and meaningful interpretation. Recognizing the improbability of the complete harmonization of protocols, we aimed to define and propose a set of best practices to maximize confidence in the data generated by laboratories. The work presented here reviews the pitfalls and limitations of the assay, proposes strategies to enhance assay sensitivity and robustness, and outlines approaches for data analysis, reporting, and interpretation. Additionally, the potential of the MAPPs assay for future applications such as clinical studies is discussed. By proposing measures and controls that support the development of high-quality MAPPs assays, we seek to improve their reproducibility and reliability for drug candidates’ nonclinical immunogenicity risk evaluation.

## 1 Publication scope

Laboratories typically create their own MAPPs assay protocols based on factors such as available cell sources, infrastructure, equipment, requirements of the specific scientific question, or additional *in vitro* assays that are run concurrently. As a result, practices can vary between different labs. Recognizing the variety of existing MAPPs assay protocols, this publication refrains from recommending a specific approach. Instead, it provides comprehensive best practices to aid scientists develop robust MAPPs assay workflows that deliver accurate results via implementation of multiple quality controls and meaningful data interpretation. This discussion will pinpoint common pitfalls and provide strategies to enhance assay sensitivity and robustness. However, recommendations will not extend to specific brands of reagents, consumables, or instruments, maintaining a focus on general best practices and methodological rigor.

## 2 Central role of MAPPs assays in drug development

**Figure 1:**
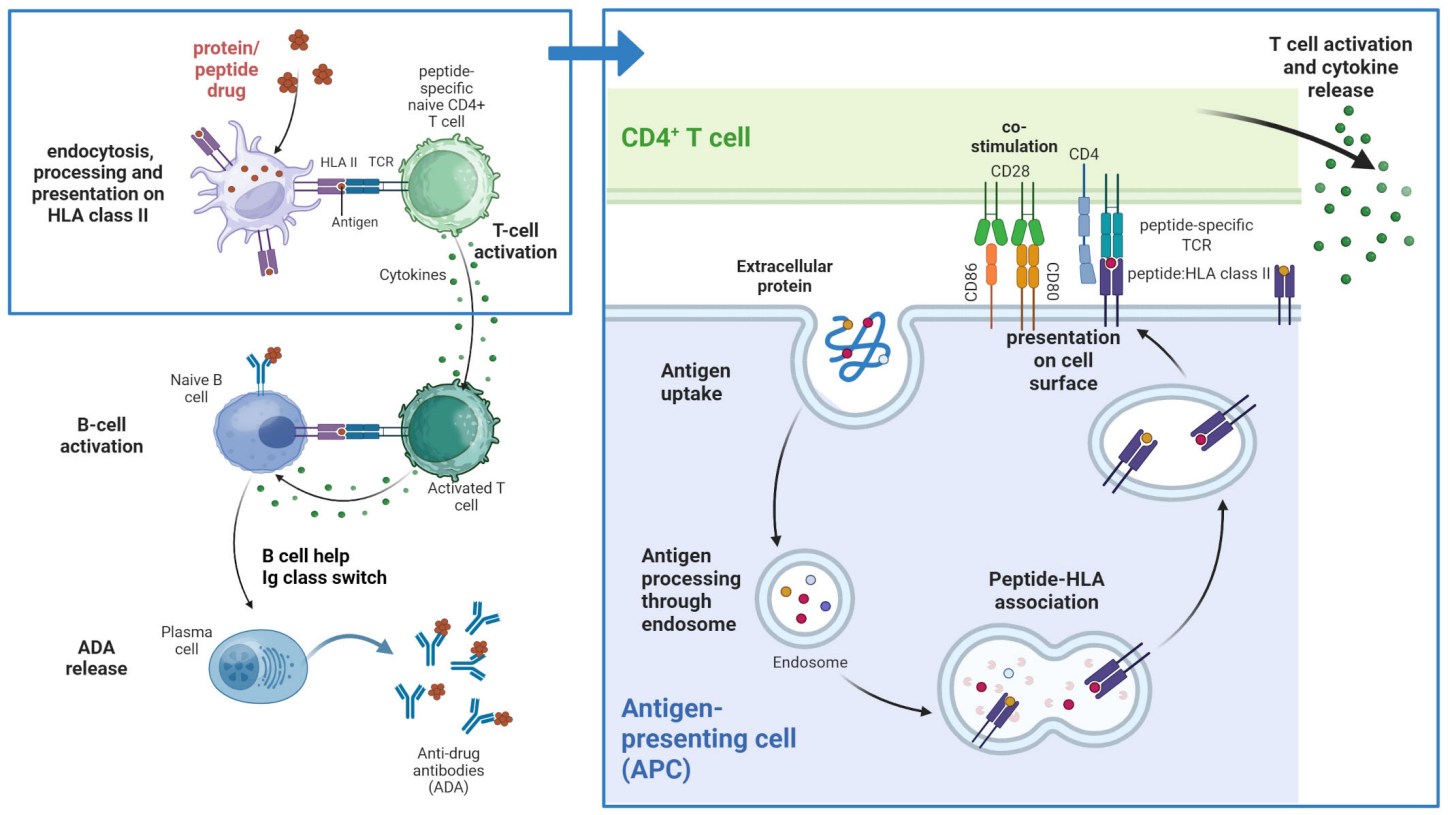
T cell-dependent immunogenicity. Peptide and protein therapeutics are taken up by professional antigen presenting cells (APCs) and processed in the endolysosome where peptides are bound to peptide-specific HLA class II molecules. The peptide:HLA complexes are presented on the surface of the APC where they can potentially be recognized by T cells with corresponding peptide-specific T cell receptors. This recognition and additional interaction of co-stimulatory molecules leads to activation of the T cell and cytokine secretion which in turn can lead to B cell activation and Ig class switch and subsequently the release of anti-drug antibodies (ADAs). Created in BioRender. Karle, A. (2025) https://BioRender.com/z1efgeo.

In its broader definition, immunogenicity is an unwanted immune reaction against an antigen which encompasses both humoral and cellular responses. In drug development, however, immunogenicity most commonly refers to the humoral component of the response, i.e., the formation of class-switched, affinity-matured anti-drug antibodies (ADA) against B cell epitopes, initiated via T cell epitopes in the amino acid sequence of the drug (**Figure 1**). ADA can be neutralizing or non-neutralizing, and both types have the capacity to affect the drug pharmacokinetic and in certain cases also the pharmacodynamic profile, which can lead to loss of treatment efficacy. Additionally, ADA-mediated adverse events can compromise the safety of the drug (1). Drug immunogenicity results from a cascade of immune cell interactions, in which drug-derived antigen presentation by professional antigen-presenting cells (APCs), such as dendritic cells (DCs), plays an essential role.

Immunogenicity risk assessment (IRA) (2) can be understood as a process that allows the establishment of effective protein design, mitigation, and monitoring strategies. The MAPPs assay is a nonclinical immunogenicity risk assessment tool for the analysis of product-related risk factors that identifies peptides having the potential to trigger a T cell response, thereby informing the risk of developing ADA based on the presence of reactive T cell epitopes in the biotherapeutic. In the context of this assay, immunogenicity *risk* is commonly referred to as immunogenicity *potential*. It is important to note that MAPPs assessments do not predict the clinical consequences to the patient associated with ADA development, but should rather be considered a piece in a broader framework puzzle for evaluating immunogenicity risk. Immunogenicity development depends on various factors such as sequence, format, critical quality attributes, and the patient’s immune status. For replacement therapy products or therapeutics utilizing human protein domains, the presence of germline or modified human protein sequences can substantially increase the risk of cross-reactivity with endogenous counterparts. Additional safety risk factors have been reviewed elsewhere (2).

To date, the risk factors contributing to the immunogenicity of a biotherapeutic in humans can be estimated nonclinically using multiple tools that replicate key elements of the immunogenicity cascade. *In vivo*, standard animal models are not reliable for predicting human ADA due to differences between human and animal immune systems (3), as animal Major Histocompatibility Complex (MHC) alleles differ from human leukocyte antigen (HLA) alleles in their preferences for amino acids in the binding groove (4, 5). Minipig (6, 7) and murine models (8, 9), transgenic for some or all components of the human immune system, have been described as an alternative, with the caveat that they are not fully tolerant to human proteins due to the differences in self-peptides presented to T cells during thymic selection. In silico, HLA class II binding prediction tools provide high throughput analysis, but often miss nuances of antigen processing within cells such as dynamic and structure-dependent protein unfolding and enzymatic degradation processes, which may lead to over- and under-predictions (10). Predicted epitopes, however, can support the interpretation of MAPPs data by defining potential core binding sequences, associated HLA haplotypes, and germline frequencies of epitopes. *In vitro*, assays using human immune cells can be employed to assess nonclinical immunogenicity risk at different steps of the immune cascade, including APC activation, antigen presentation, and CD4 T cell responses. These are valuable tools to assess the sequence- and format-associated factors of protein-based therapeutics that may contribute to the development of immunogenicity, and the confidence in these assays increases as they undergo continuous improvement.

The principles behind the MAPPs assay were developed in the 1990s, initially to understand the mechanisms of peptide presentation (11, 12) and identify tumor antigens (13). It was further proposed as a tool for nonclinical immunogenicity risk assessment (14) and used to inform molecular design (15, 16), candidate selection (17), and mechanistic investigations (18–25). Health authorities recommend that sponsors conduct a structured immunogenicity risk assessment to evaluate the potential for anti-drug antibody (ADA) formation and its clinical consequences (2). While current regulatory guidelines do not mandate the routine execution of preclinical in silico or *in vitro* assays such as MAPPs, they strongly encourage their use when justified by the risk profile of the molecule. Consequently, companies are expected to design a scientifically sound and risk-informed strategy that integrates mechanistic understanding, molecule-specific attributes, and clinical context. This strategy should guide the selection and timing of preclinical assays and tools (26). In cases where the MAPPs assay has been performed and the data is relevant to the submission of a given biotherapeutic, its outcome should be summarized in the Integrated Summary of Immunogenicity (ISI). *In vitro* assays, as part of the nonclinical immunogenicity risk assessment toolbox, have distinct benefits and limitations as they survey various cellular aspects of the immunogenicity cascade (3). The MAPPs assay, when properly established, is a highly reliable method that identifies amino acid sequences that may induce immunogenicity of biotherapeutics. Thus, using this assay early in the development process is advantageous to identify hotspots within the sequence and may offer substantial optimization potential for biotherapeutics (27) that otherwise may bear immunogenic risks (28). MAPPs assays are also invaluable for ranking drug candidates based on their intrinsic estimated immunogenicity potential (29), thus aiding in the selection of better development candidates. Furthermore, they facilitate mechanistic investigations into the impact of aggregates (30), post-translational modifications (31), target engagement (32) and mutations. Protein fusions, glycosylation (33) and other modifications may impact how professional APCs internalize, digest and process the proteins to peptides, and their impact on the peptide patterns can be assessed. MAPPs data can be used to train and improve in silico tools (34), providing an improved framework for de-immunization approaches, particularly for molecules with a known or expected likelihood for immunogenicity. For applications aiming at reducing protein immunogenicity during the design phase, identification of the 9-mer HLA-binding core via alignment of MAPPs results and in silico prediction of peptide binding can help to identify positions where amino acid substitutions can abolish HLA-binding.

The MAPPs assay described and discussed here is performed with the intent of identifying HLA class II-associated peptides. Albeit less frequently reported, the MAPPs assay principles can also be applied to the characterization of HLA class I associated peptides, by simply using a reagent that immunoprecipitates peptide-HLA class I receptor complexes rather than one targeting HLA class II. This is of particular interest for the de-immunization of specific drug products, for example nucleic acid-encoded protein that is translated intracellularly *in vivo*. In such a case, intracellularly produced proteins follow the HLA class I presentation pathway during degradation, with derived peptides potentially leading to unwanted CD8 cytotoxic T cell responses. Examples include de-immunization of the viral capsid protein in adenovirus associated virus-based gene therapy (35) and de-immunization of bacterial nucleases in gene editing therapy products (16).

## 3 Clinical relevance

Several publications have demonstrated the biological relevance of the peptides identified using MAPPs assays. Studies show that HLA-II associated peptides presented by monocyte-derived dendritic cells (moDCs) align well with genuine reactive T cell epitopes found in healthy individuals and in patients who have developed an ADA response against a drug (20, 22, 36–39). The correlation between MAPPs and T cell activation assays emphasizes the importance MAPPs assays have in identifying potentially immunogenic regions of therapeutic proteins. However, it is important to note that MAPPs assay results, on their own, cannot accurately predict but only estimate clinical immunogenicity incidence. Currently, MAPPs assays are almost exclusively conducted using samples collected from randomly selected or HLA pre-typed healthy volunteers. To date, there have been no reports demonstrating a direct causal link within an amino acid sequence derived from a biotherapeutic identified by MAPPs and the occurrence of an ADA event in the same patient. Apart from regulatory challenges to obtain samples from such patients, the requirement for large numbers of high quality cells and the high sensitivity needed to measure the low amounts of the drug’s presented HLA-II associated peptides are formidable obstacles to enable such measurements. Furthermore, a separate T cell activation assay (preferably using T cells isolated from the same individual) is also needed to evaluate the likelihood of a MAPPs-identified peptide causing an immune response. In addition, the clonal analysis of immortalized B cells reactive to a specific biotherapeutic may also shed light on specific amino acid sequences at higher risk of immunogenicity (38). Nevertheless, with improvements in assay sensitivity a more systematic and generalized approach using the MAPPs assay to survey large numbers of patients enrolled in clinical trials may enable statistical approaches to correlate the detection of immunodominant biotherapeutic-derived HLA-II associated peptides with the development of ADA in genotyped patients. Such an endeavor, however, will require standard operating procedures, systematic and regulated sample collection procedures, and the use of generic analytical and reporting protocols.

## 4 Recommendations for robust assay results

### 4.1 Short description of the MAPPs assay

**Figure 2:**
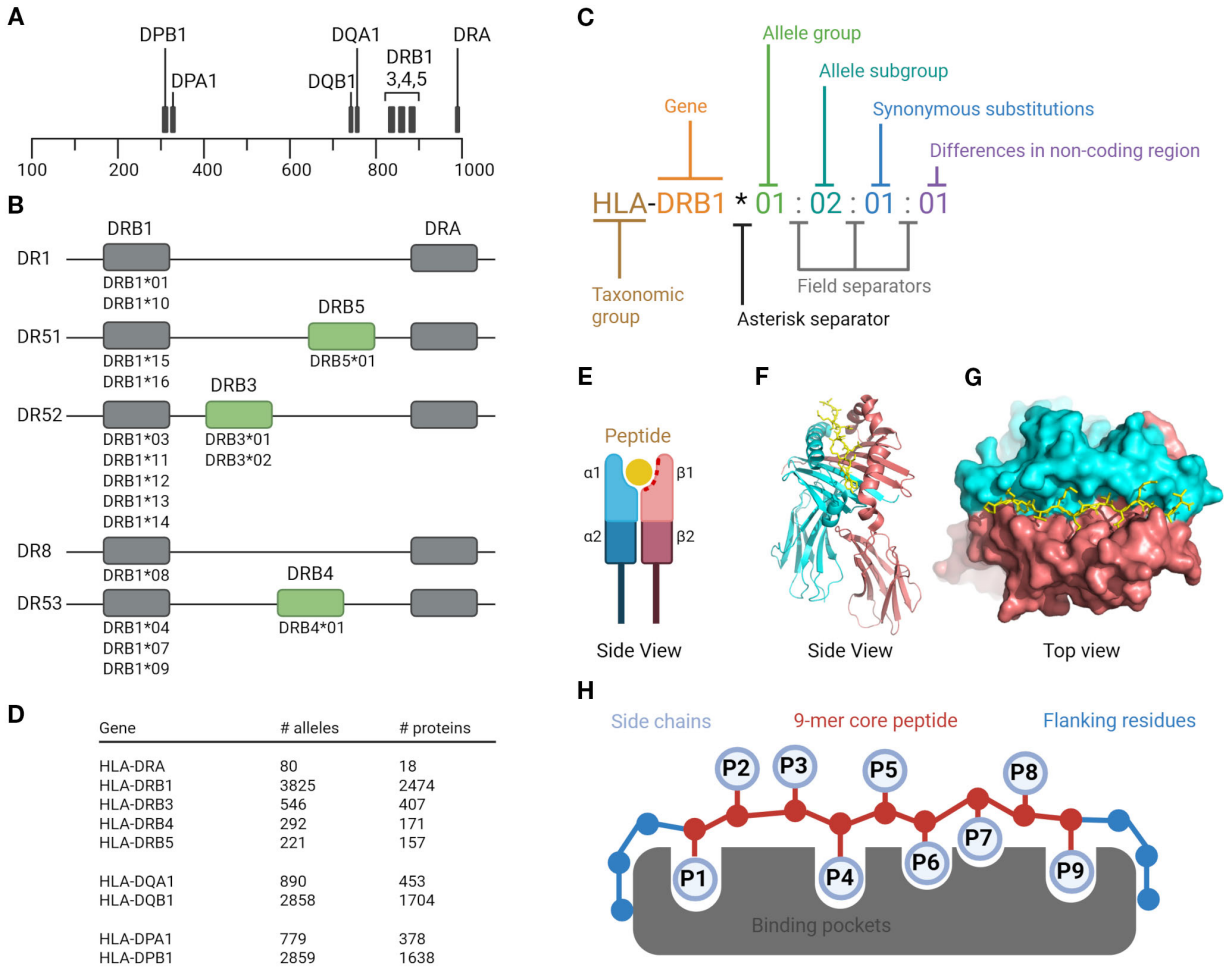
Human leukocyte antigen class II system. HLA class II molecules are composed of three types: two HLA-DR, one HLA-DQ, and one HLA-DP molecule per haplotype. Each molecule consists of a heterodimer formed by one α (alpha) and one β (beta) chain; **(A)** Gene localization of HLA class II. The DPA, DQA, and DRA genes encode the α chains, while the DPB, DQB, and DRB genes encode the β chains. The physical proximity of the DQ and DR gene loci contributes to linkage disequilibrium, resulting in frequent allelic associations between HLA-DQ and HLA-DR (e.g., HLA-DR15/HLA-DQ6); **(B)** Haplotype structure of HLA-DRB genes. Each haplotype expresses one HLA-DRB1 gene and, depending on the allele present at this locus, may also include an additional DRB3, DRB4, or DRB5 gene. These genes encode structurally similar but genetically distinct β chains. Importantly, the strong linkage disequilibrium between HLA-DRB1 and these additional DRB genes are highly stable across populations. For example, DRB1*15 is almost always co-inherited with DRB5, forming the DR15/DRB5 haplotype, while DRB1*03 is associated with DRB3, and DRB1*04 with DRB4; **(C)** Nomenclature of HLA; **(D)** HLA polymorphism and number of alleles and proteins (IPD-IMGT/HLA-database); **(E)** HLA class II molecule structure schematic view. The peptide-binding site is formed by the α1 and β1 domains of the α and β chains; **(F)** Side view of the crystal structure for HLA-DR1 (PDB ID 1DLH) shown in cartoon representation. The α chain is colored red and the β chain cyan; **(G)** Top view of the same structure shown in surface representation. The HA 306–318 peptide is depicted in stick representation. The α and β chains are shown in red and cyan, respectively; **(H)** Peptide binding mode. Amino acid side chains of anchor residues P1, P4, P6, P7 and P9 interact with corresponding binding pockets in the peptide binding groove. Created in BioRender. Karle, **(A)** (2025) https://BioRender.com/eb39tt4.

**Figure 3:**
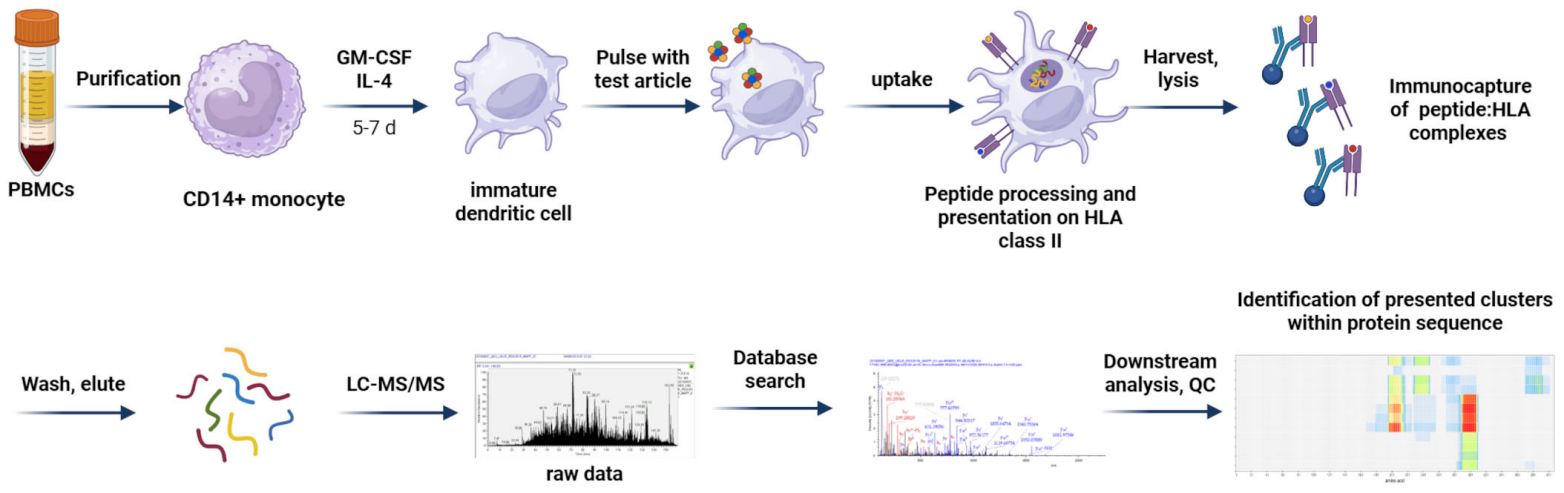
MAPPs assay workflow. CD14+ monocytes are isolated from PBMCs of healthy donors and differentiated into moDCs. Immature moDCs are pulsed with test articles, which are internalized and processed. Test article-derived peptides are presented on HLA class II on the cell surface. After harvest and lysis of the cells, HLA:peptide complexes are captured and washed; HLA-bound peptides are eluted and subjected to LC-MS/MS analysis. MS spectra are matched against a protein database and test article-derived sequences are mapped to the protein of interest allowing identification of presented clusters within the protein sequence. Created in BioRender. Karle, A. (2025) https://BioRender.com/hykimx2.

The MAPPs assay is a method used to identify peptides presented by HLA class II molecules (**Figure 2**) on APCs using liquid chromatography-tandem mass spectrometry (LC-MS/MS). The output obtained from a MAPPs assay, a list of peptides, is highly dependent on the APC cell type and HLA allotype, assay conditions, and the analytical methods used to identify these peptides. Therefore, we have divided our discussion of the MAPPs assay into two sections: a biological, “wet lab” component, (described in section 4.2-4.5 below) where a test compound is assayed in a cellular assay to produce HLA-II-associated peptides that are immune-enriched for subsequent analysis; and an analytical, “technical” component (described in sections 4.6-4.8 below) where the isolated HLA-II peptides are analyzed using LC-MS/MS and results are reported back to the user using specialized software packages. An overview of the full assay workflow is illustrated in **Figure 3**.

### 4.2 Donors and cell types

MAPPs assays are typically performed using moDCs differentiated from monocytes obtained from healthy donors (20). Monocytes are readily obtained from donor whole blood, buffy coats, or leukopaks, providing a reliable source of cells for the assay. MoDCs efficiently internalize and proteolytically process exogenous proteins and present the resulting peptides on HLA class II molecules (**Figure 2**). HLA II molecules are highly polymorphic (**Figures 2A–D**) (40), therefore, when a clear HLA association of the targeted disease is known, the donor set should ideally be representative of the HLA diversity of the target patient population, i.e., the allele distribution in the donor set should match that of the population of reference (41). Of note, the matching is commonly based on HLA-DRB1 alleles only, while it is becoming more evident that other HLA Class II alleles (**Figure 2A**) are playing a relevant role as well (42–44). Peptides that bind to HLA class II alleles can vary in length due to the open binding groove (**Figures 2E–G**). HLA-DRB1 alleles contain binding pockets at positions 1, 4, 6, 7, and 9 within the binding core (**Figure 2H**), and peptide binding is influenced by the preferences of specific amino acids in these pockets. Additionally, the flanking regions may contribute to peptide binding (45, 46).

In some cases, attention should also be paid to alleles that have been associated with ADA development (47, 48). If donors are pre-typed, as few as 12–15 donors can be sufficient to represent the most frequent HLA alleles, whereas higher numbers of donors may be required for donors not previously HLA typed. EBV-transformed B cell lines are another potential source of cells for MAPPs assays (27), however, care must be taken to establish that antigen uptake and processing is similar to that of cells obtained ex vivo and that the HLA diversity of the target population is represented. B cells also efficiently process and present exogenous peptide antigens on HLA class II molecules. In contrast to moDCs, B cell antigen uptake is primarily mediated by the antigen-specific B cell receptor (49, 50). The low frequency of B cells specific for a given target antigen must therefore be considered when developing a B cell-based MAPPs assay.

### 4.3 Cell quality control and culture conditions

As with all cell-based assays, high quality cells, along with appropriate cell quality controls and cell culture conditions are essential to achieving robust and reproducible MAPPs assay results. Peripheral blood mononuclear cells (PBMCs) are typically isolated from buffy coats or leukopaks. Individual labs have standardized isolation processes, but approaches can vary between labs. Various manual and automated protocols exist, and a typical manual method performed is density gradient centrifugation. Buffy coat cells are diluted and then carefully layered onto a density gradient medium (DGM). The sample is centrifuged which creates a layer of PBMCs at the interface with the DGM and subsequently the PBMCs are carefully extracted and washed (a similar process can also be followed for leukopaks and whole blood). The PBMCs recovered should be analyzed by flow cytometry for quality, viability, and to verify that sub-populations (e.g., monocytes, T cells etc.) are within expected ranges. PBMCs can then be used for monocyte isolation in their fresh state or can be cryopreserved for subsequent use. When opting for cryopreservation, an optimized protocol and cell viability test post-thawing are imperative to ensure cellular integrity and functionality.

CD14+ monocytes are commonly isolated from PBMCs utilizing magnetic beads and many different brands are available with either positive or negative selection protocols. For quality control, it is important to characterize monocyte purity and phenotype after isolation via flow cytometry (**Table 1**). Monocytes should be highly viable (>90%), pure (percent CD14+ >90), high in CD14 expression with and low side scatter. Most laboratories in the EIP consortium differentiate monocytes to immature moDCs maintaining a cell density between 3E5-1E6 per mL. Assay medium containing 10% fetal bovine serum (FBS) is commonly used with success but often requires testing of different FBS batches as cell viability and antigen uptake may potentially vary. Human AB blood-type serum containing media and serum-free media have been applied successfully across different EIP labs as well, although differences in peptide presentation efficiency have been observed with the latter in comparison to FBS-containing media. Competition experiments indicate that high concentrations of Intravenous Immunoglobulin (IVIG) do not interfere with drug-peptide presentation (51), suggesting that proteins from FBS should not meaningfully compete with and impact the drug-derived antigen presentation profile. Culture medium should contain granulocyte-macrophage colony-stimulating factor (GM-CSF) and interleukin 4 (IL-4), both of which should be titrated to achieve fully differentiated moDCs. Medium does not require exchange during the differentiation phase, but some EIP labs replenish GM-CSF and IL-4 after a few days of cell culture. By ensuring that the differentiated immature moDCs are of high quality and properly characterized, researchers can obtain more reliable and reproducible results from their MAPPs assays. On the day of stimulation, commonly between days 5 and 7, the differentiation status must be verified via flow cytometry (**Table 2**). Viability should exceed a lower limit of 70% viability after excluding debris. Upon differentiation, immature moDCs should be low to negative in CD14. Moreover, a visible (positive) shift in forward/side scatter of cells should be observable, and cells should express CD11c and CD209 as classical DC markers. Immature cells should be low in expression of activation markers e.g., CD80, CD83, CD86, to ensure that cells are able to efficiently take up proteins from their surroundings, process them to peptides and present the peptides on HLA class II molecules. Pre-activated moDCs typically yield fewer drug-derived peptides and lead to overall lower assay sensitivity.

**Table 1:** Flow cytometry panel for monocyte purity.

Surface marker		Monocytes
CD14	essential	pos
Live/Dead stain	essential	neg
CD3	optional	neg
CD19	optional	neg
CD56	optional	neg

**Table 2:** Flow cytometry markers for DC differentiation and activation.

Surface marker		Immature moDC (differentiated)	Mature moDC (activated; if maturation stimulus used)
Live/Dead stain	essential	neg	neg
CD14	essential	Low	Low
HLA-DR	essential	High	Very high
CD209	Essential; one sufficient	Very high	High
CD11c		High	High
CD40	optional	High	High
CD80	essential; one sufficient	Low	High
CD83		Low	High
CD86		Low	High

Incubation duration with the test article is another key assay factor, with 24 hours being preferred by EIP labs to minimize cell death and enhance signal quality, although longer durations, up to 72 hours, may improve the identification of peptides (A. Ducret, A.C. Karle, M. Gutknecht, personal communication) with higher affinity and may be considered for specific applications. Most EIP labs use between 5E5 and 5E6 moDCs to generate a single MAPPs sample. Consistent volume addition to the cells should be ensured across different test compounds to avoid different end concentrations in the cell culture. Compounds can be pre-diluted in phosphate buffered saline (PBS) or moDC cell culture medium. When adding test compounds to the cells, culture volume increases of more than 20% should be avoided to maintain stable cell culture conditions. It is also common for the immature moDCs to be activated/matured with 0.1-1 µg/mL of LPS or a cytokine cocktail during incubation of the cells, to increase the number of HLA:peptide complexes on the cell surface (and closer represent the moDC that may be used in a T cell co-culture assay). The activation cocktail should be optimized to ensure sufficient moDC maturation without impacting the viability and functionality of the moDCs. The timing of activation cocktail addition to the immature moDC should also be optimized (e.g., should it be added at the same time as the compound or after a defined period) as well as the duration of activation (e.g., 24–72 hours). Ideally, the cells should be reassessed via flow cytometry on the day of harvest as this may reveal potential drug-associated toxicity or interference with maturation. In case moDCs are loaded with the test article in plates, it can be helpful to count cells after harvesting to enable normalization of identified peptides per sample. Test-article loading of moDCs in tubes can facilitate harvesting, ensure standardization of cell numbers across samples, and increase assay robustness.

### 4.4 Test article concentration

Test article concentration is an important variable in the MAPPs assay. Concentrations required for the sensitive detection of the test article-derived peptides should be empirically determined for the scope of the assay (see below) using a titration design (52), noting that the optimal concentration for sensitive assay results may not necessarily be equivalent to the *in vivo* concentration in blood or tissues. Protein should be added in molar concentrations to ensure consistency across test articles of different molecular weights. The partners in the EIP consortium typically dose moDCs at concentrations between 0.3 µM and 4 µM in the final cell culture.

Using insufficient test article concentration may yield drug-derived peptides below detection, leading to false negative results. Alternatively, excessive concentration can result in an increased number of peptide copies without necessarily leading to an increase in the number of identified clusters (sets of peptides of varied lengths that share a common core sequence, 53). This can lead to saturation of the MS and a narrower dynamic range of the assay. High numbers of identified peptides can also complicate the process of assigning individual peptides to overlapping clusters while not providing relevant additional information. During assay implementation, test article concentrations should therefore be carefully optimized via titration (52) for the specific application. For de-immunization purposes, undetected peptide clusters can be detrimental, hence high concentrations need to be used to ensure high sensitivity detection. In contrast, for candidate ranking, intermediate concentrations may be more effective and practical. Although not every cluster may be detected in such a case, differences between candidates may be easier to discriminate due to a higher dynamic range for peptide detection below saturation concentrations in the MS, based on the employed LC-MS peptide separation and detection method.

### 4.5 Cell lysis and peptide isolation

After harvesting the drug-treated moDCs, cells are typically separated from medium via centrifugation and washed with PBS to remove media components. Often, the resulting cell pellet is frozen and subsequently lysed in a buffer that contains protease inhibitors and detergent to enable solubilization and extraction of proteins from membranes. Alternatively, cells can first be lysed and then separated from the cell debris pellet by centrifugation, after which the lysate can be frozen. The chosen detergent should efficiently extract HLA proteins while maintaining intact HLA-peptide complexes. Triton X-100 was used historically, but new detergents such as CHAPS with lower toxicity are now commonly used.

Immunoprecipitation of HLA class II-peptide complexes in MAPPs assays must take into consideration the specificity of the capture antibody. HLA class II molecules include HLA-DP, HLA-DQ, and HLA-DR heterodimeric proteins each comprising an alpha chain and a beta chain. The HLA-DR molecule is more complex and comprises the alpha chain from the HLA-DRA locus and up to four different beta chains HLA-DRB1, -DRB3, -DRB4, and -DRB5 which only appear in certain combinations (**Figure 2B**). HLA-DRB1 alleles are highly polymorphic (**Figure 2D**), and the majority of the population is heterozygous, comprising two different alleles differing in their binding preferences of amino acids at anchor positions. An individual can therefore display peptides from up to 4 different HLA-DR alleles, two different HLA-DRB1 alleles, each paired with either of HLA-DRB3, -DRB4, or -DRB5 alleles. Monoclonal antibodies capturing different HLA haplotypes can be produced in sufficient quantity and have low batch to batch variability, which makes them the reagent of choice. Antibody clones are available for HLA-DR, the predominant HLA class II protein, as well as pan-class II which binds to HLA-DP, HLA-DQ, and HLA-DR. The pan anti-HLA-DR antibody clone L243 (54) recognizes HLA-DRB1, DRB3, DRB4, DRB5 alleles paired with the DRA alpha chain (**Figure 2B**). However, antibodies recognizing a broader set of alleles may become of interest (clones IVA12, CR3/43, WR18, Tü39, HB-145) for therapeutics with known HLA allele associations. However, the binding epitopes of these antibodies are often not fully elucidated, and they may not comprehensively cover all HLA class II alleles. In addition, antibodies specific to HLA-DP (clone B7/21) or HLA-DQ (SPV-L3 and 1a3) are available and may be of interest for specific investigations. The choice of capture antibodies in the MAPPs assay depends on the given application and key scientific questions to address. The impact on cost and complexity for routine use should also be taken into consideration. Several options are available for the immunoprecipitation step. The simplest method is the addition of the antibody or mixture of antibodies of choice in the cell lysate, followed by incubation for 30–60 minutes at room temperature or overnight at 4 °C, and capture of the antibody with protein-A or protein-G coated beads. Alternatively, antibodies can be covalently bound to Sepharose-4B beads or NHS-coated magnetic particles. Recently, many commercially available antibody clones have become available in a biotinylated version, which enables their efficient capture using avidin-based chemistry. Using immobilized antibodies facilitates the use of robotic pipetting systems, which can greatly increase throughput and consistency.

After immunoprecipitation, HLA class II-peptide complexes are washed with a detergent-containing buffer, usually the same buffer used at the lysis step, to remove lipids and non-specifically bound proteins, followed by extensive washing with buffer or water to remove detergents and contaminants that could interfere with the MS analysis. It is recommended to optimize the washing steps so that minimal detergent-derived signals are detectable. Immunoprecipitates are then acid eluted to recover peptides. During peptide isolation and purification, it is crucial to minimize the number of processing steps to reduce non-specific losses to surfaces. Exposure to large surfaces and materials that introduce leachates, which could interfere with MS analysis, should be avoided. Testing different brands of plasticware and pre-rinsing with the buffer or solvent used in the process can also help mitigate these issues. Of note, low protein binding plasticware may prolong the evaporation process due to increased surface tension. During sample drying, high volumes and exposure to elevated temperatures should be avoided to prevent peptide losses and degradation. Careful application of these steps will enhance the purity and integrity of the isolated peptides.

### 4.6 LC-MS measurement

A full description of possible LC-MS/MS equipment is beyond the scope of this paper and the reader may consult numerous recent publications (for example, (21, 32, 55, 56) on how to best perform this step according to the equipment at hand. Typically, due to the required sensitivity and the limited sample amount, nanoflow LC-MS systems (operating in the nL/min range and using nanoflow chromatographic columns with inner diameter of 100 μm or below) are employed. Additionally, implementing systems that purify the air at the MS inlet can help reduce airborne contaminants that may interfere with peptide detection. The use of in-line filters and trap columns can also enhance assay robustness, allowing for the analysis of more samples on the same analytical column while increasing data quality by sharpening peaks and removing contaminants and small debris. Newer model mass spectrometer instruments will typically provide higher sensitivity and, therefore, a broader peptide mapping capability of the test article. The ability to measure MHC-II peptides using a high-resolution mass analyzer (< 30 ppm) both in full scan and fragmentation mode will provide additional confidence in correctly identifying MHC-II peptides (see also below and section 4.8).

The full or partial MAPPs sample can be injected for a given analysis. For analysis, isolated peptides are usually resolubilized in a minimal volume of sample buffer containing 2-5% acetonitrile and 0.1% formic acid and chromatographically separated on a reversed phase column with a 5%–80% acetonitrile/0.1% formic acid gradient with a throughput of typically 30 to 60 min per injection. The first part of the gradient (up to around 40% acetonitrile content) usually yields most HLA class II peptide identifications while the remaining gradient serves as a column cleaning step before column equilibration. Gradient slope particularly in the first part of the gradient can be adjusted to match the mass spectrometer acquisition rate to ensure adequate sampling of the eluted peptides. There are a variety of chromatography column types, lengths, and brands with C_18_ or similar materials that can be selected based on the specific needs.

The large majority of mass spectrometers used in MAPPs applications are operated in data-dependent acquisition mode (DDA), due to the relatively simple complexity of the peptide mixture (typically, 2,000-5,000 total peptides identified per LC-MS run) and a straight-forward data interpretation (e.g., (57). In this mode of operation, the mass spectrometer first acquires a full mass survey scan (MS1) that is used to select 5–20 of the most intense signals for fragmentation (MS2). It is advisable to use dynamic exclusion, typically 10 to 30 seconds in duration depending on the LC peak widths and acquisition rate of the instrument and specific application, to reduce repeated analysis of highly abundant peptides and improve detection of low abundance peptide peaks. In particular, DDA is a preferred method to identify peptides from drugs for which there has not been prior analysis using MAPPs. Recently, some researchers have argued that mass spectrometers operated in data-independent acquisition (DIA) mode, a newer proteomic approach that fragments all precursor ions within pre-defined isolation windows (58), might become a superior analytical strategy for MAPPs. One of the principal advantages of DIA-MS lies in its remarkable reproducibility of peptide identification across numerous experiments. In this mode of operation, study-specific spectral libraries are typically created from in-depth DDA analysis of samples before proceeding with DIA-MS analysis. However, a pre-study is necessary to generate the drug-specific spectral library prior to analyzing the study samples, therefore DIA-MS is mostly useful for studies on recurring molecules. Library-free DIA approaches are an alternative to experimental spectral libraries but must be benchmarked to DDA analyses (see also section 4.8 for a database searching discussion).

### 4.7 Assay quality controls

As with any assay reporting relative values (the number/intensity of peptides/clusters identified within an assay using a defined number of moDCs), it is imperative to include quality and functional controls to demonstrate assay performance using known standards. Several assay controls should be included: i) unchallenged samples for each donor to evaluate whether drug-derived peptides detected in treated samples could be false positives in case of partial sequence identity with other proteins; ii) an internal protein control such as a well-presented endogenous proteins (**Table 3**) to verify an expected antigen presentation pattern and intensity; iii) at least one protein with known antigen presentation pattern needs to be used as cell functionality control, to confirm adequate antigen uptake and presentation. Ovalbumin, the major birch pollen allergen Betv1a, or commercially available monoclonal antibodies (mAbs) are frequently utilized for this purpose. A list of well-presented peptides from infliximab and rituximab are summarized in (**Table 4**) which can support labs in estimating their assay sensitivity. If moDCs are cultured in FBS-supplemented media, a bovine protein can also be considered. Finally, if the scope of the assay is to compare different compounds, it is of importance to carry out the study at the donor level (that is, to compare each test article side-by-side in each donor) to ameliorate the variability arising from donor-dependent processing and presentation of each moDC preparation.

**Table 3:** Examples of endogenous proteins and media components well-presented by moDCs.

Source	Abbreviation	UniProtKB entry	Full protein name
Human	ITGAM	P11215	Integrin alpha-M
Human	DRA	P01903	HLA class II histocompatibility antigen, DR alpha chain
Human	GAPDH	P04406	Glyceraldehyde-3-phosphate dehydrogenase
Human	VIM	P08670	Vimentin
Human	COL1A1	P02452	Collagen alpha-1(I) chain
Human	AMPN	P15144	Aminopeptidase N
Human	SDCBP	O00560	Syntenin-1
Human	HLAB	P01889	HLA class I histocompatibility antigen, B alpha chain
Human	CATZ	Q9UBR2	Cathepsin Z
Human	MA2B1	Q93093	Lysosomal alpha mannosidase
Human	HG2A	P04233	HLA class II histocompatibility antigen gamma chain
Human	ANXA2	P07355	Annexin A2
Human	TFR1	P02786	Transferrin receptor protein 1
Human	MCR1	P22897	Macrophage Mannose receptor 1
Human	ALBU	P02768	Human serum albumin
Bovine	APOB	E1BNR0	Apolipoprotein B

**Table 4:** Frequently observed sequence regions in infliximab and rituximab; Peptides summarized that bind to 15% of tested donors or above.

Compound	Chain	# of donors with cluster	Sequence region
infliximab	Heavy chain	5/34	SPEKGLEWVAEIRSKSINSATHYAE
		17/34	ISRDDSKSAVYLQMTDLRTEDTG
		5/34	TEDTGVYYCSRNYYGSTY
	Light chain	15/34	TQSPAILSVSPGERVSFS
		8/34	GSPRLLIKYASESMSGIPS
rituximab	Heavy chain	13/34	ADKSSSTAYMQLSSLTSEDSA
	Light chain	8/34	SQSPAILSASPGEKVT
		18/34	KPGSSPKPWIYATSNLASGVPV
		6/34	SGSGTSYSLTISRVEAEDA

For ranking purposes, it can be helpful to include sensitivity controls, biologically relevant molecules with known assay response profiles in terms of the pattern and frequency of clusters presented by moDCs. Even though they may not be fully comparable to the test articles due to sequence differences, they can serve as guides to bracket the responses observed. Biotherapeutics show differences in the internalization rate by APCs, their enzymatic processing, and the binding ability of biotherapeutic peptides to HLA class II molecules. As a result, they show differences in presented peptide- and cluster frequencies, which can be considered as lower or higher antigen presentation. For mAbs, adalimumab and infliximab have been used by EIP labs as a high antigen presentation control and trastuzumab or ustekinumab as low antigen presentation controls. For other modalities, there is a general lack of well characterized high and low antigen presentation controls. In case sensitivity controls are used that have been well characterized regarding their antigen presentation pattern, they can replace the cell functionality control. Although not always available and well characterized, additional molecules with the same format and length as the test article can be considered if such a comparison is deemed important for a given project.

The same level of care is of utmost importance regarding controlling the performance of the LC-MS/MS instrumentation, including a chromatographic system suitability standard and a low-level peptide identification standard (such as HeLa digests), to demonstrate a broad and reproducible elution profile across hydrophilic and hydrophobic peptides and to track the instrument sensitivity and calibration. For meaningful data interpretation, the total number of identified peptides, accounting for multiple detections, should surpass 400 as the lowest threshold (using standard MS equipment; this number might be considerably higher, above 1000, using latest generation instrumentation). Ideally, unique identified peptides should exceed 1000, and total identified peptides should exceed 1500. It is important that the total and unique peptide counts remain comparable across different drugs tested on the same donor, with a variance of no more than 30% between runs. Donors with high sample-to-sample variability should be excluded in case the data is used for candidate ranking purposes. This rigorous approach ensures the reliability and robustness of the peptide analysis, facilitating the accurate assessment of antigen presentation and drug efficacy.

Two additional quality control measures should be included, at a minimum during assay implementation for good practice, but ideally for each batch of beads or columns produced. 1) A western blot to assess HLA solubilization and pull-down efficiency or alternatively LC-MS, comparing the peptides isolated using the new batch against those via previous batches and 2) A histogram representing size distribution of identified peptides for each sample to ensure the length distribution corresponding to HLA class II derived peptides (majority between 9 and 20 amino acids peaking between 15 and 17 amino acids).

### 4.8 Data analysis, reporting, and interpretation

A full description of all available search engines is beyond the scope of this paper; however, while most packages will deliver appropriate results, data interpretation will be facilitated using a peptidomics rather than a conventional proteomics approach (the “end product” is an MHC-II peptide associated to an HLA molecule). MAPPs MS data acquired either in DDA or DIA mode is typically analyzed using algorithms that match the experimental fragmentation spectra against theoretically generated spectra from a provided protein database. This database should include the amino acid sequence of the test article, a human protein background (usually the full human proteome), and other non-human protein entries to enhance detection confidence and reduce false positives. For example, commonly identified bovine protein sequences should be included if bovine serum is used for moDC culture. The database search should include common post-translational modifications such as oxidation of methionine, deamidation of asparagine and glutamine, and pyroglutamate (59) as they can occur during cell culture or sample handling. Accordingly, due to the individual identification of each peptide, it is good practice to confidently call a post-translational modification only with the co-identification of the unmodified peptide species, as the determining fragment ion(s) might be missing in the spectrum due to the uncharacteristic fragmentation pattern of MHC-II peptides. As the database searching is performed without cleavage specificity (that is, the peptide cleavage site is considered random), it is important to note that the addition of dynamic modifications will considerably slow down the search speed as they substantially increase the search space. The no-enzyme database search should include a range of peptide lengths of 10 to 30 or even up to 40 amino acids to allow the identification of classical HLA-II presented peptides without greatly expanding the search space. In a peptidomics approach, results are filtered at the peptide level taking advantage of a stringent mass accuracy filtering (typically, the peptide and fragment mass accuracy is set at ± 10 ppm and ± 25 mDa, respectively, depending on the mass spectrometer instrumentation). Most EIP labs set analysis parameters to achieve a low false discovery rate (FDR) of 1% or similar using a target-decoy approach (60). The database search results are typically summarized by peptide sequence (rather than protein identification) as the data summarizes the full HLA class II receptor interactome in the sample. New *de novo* sequencing algorithms may determine the sequence of peptides directly based on the fragmentation spectra. Because these algorithms avoid sequence database matching, they eliminate the risk of data misinterpretation that could occur if the sequence of the test articles were not included in the database. However, these algorithms tend to generate fewer peptide sequence identifications due to the uncharacteristic fragmentation pattern of HLA-II peptides. *De novo* analysis can be used in conjunction with database searching to add additional specificity by using *de novo* sequence tags to reduce the protein database to those sequences with homology to identified sequence tags (61).

Data visualization usually consists of aligning identified peptides with the parental protein sequence, grouping peptides with the same core sequence into clusters, which are then considered potential epitopes for recognition by T cells. Clusters are visualized in heatmaps in which the color intensity is based on the number and/or the ion current intensity of underlying peptides within a cluster (52). When comparing compounds, the number of total and unique clusters identified for the test articles across the donor set can be used to rank the test articles. For antibody-based constructs, clusters may be reported for the full molecule, sub-domains, or those overlapping complementarity-determining regions (CDRs). Clusters within or overlapping the CDRs are generally considered to have a higher potential for immunogenicity (62) as they usually differ from common human germline sequences (36). Nonetheless, fully human sequences can still provoke immunogenicity, albeit with a typically low precursor rate of responding T cells (37). This might result in a delayed or circumstance-specific immunogenic response influenced by inflammatory immune status, degradation, aggregation, and PTMs of the drug. Reporting both unique and total clusters across the entire drug and in addition reporting cluster numbers in CDR regions for antibodies in a weighted fashion may yield optimal candidate ranking. Peptides located within or at the junctions of CDRs, mutated antibody framework or constant region sequences, or mutated sequences within therapeutic proteins should be given more weight in candidate assessment, compared to those within fully germline antibody or human protein sequences. Regions presented across multiple donors deserve particular attention. These hotspots are created by peptides that can bind multiple HLA-II alleles and therefore have the potential to impact a substantial proportion of the patient population, should they elicit a T cell response.

### 4.9 General technical limitations and considerations

MAPPs analysis provides valuable, sequence-specific data which can help guide de-immunization efforts. However, it is crucial to consider some limitations of the assay. These include, for instance, potential lack of sensitivity, relatively low throughput (see also next section), and high cell numbers and drug concentration required to allow the detection of relevant sequences (reviewed in detail elsewhere in 20). In particular, the difficulty of surveying low levels of post-translational modifications (expected or unexpected) or measuring the effect of aggregates, host cell proteins (HCPs), and other critical quality attributes (CQAs) in the drug substance using the MAPPs assay may lead to potential misinterpretation of results and associated risk. For CQAs present at low levels, for instance, the LC-MS/MS profile should be first characterized using a purified standard and the assay should be applied on isolated or CQA-enriched fractions rather than on the full drug substance and assessed using different concentrations. While, currently, MAPPs assays on enriched fractions may not yet directly determine safe CQA concentrations for patients, these mechanistic studies may prove valuable in qualitatively identifying CQAs with potential intrinsic risks thereby enabling follow-up investigations. Such information may guide technical and process development efforts to reduce the respective CQA levels.

Another point of consideration is whether a specific HLA-II peptide is expected to be detected (or not) in a MAPPs assay when the results are compared to what is predicted using popular and broadly applied *in silico* tools, such as NetMHCIIpan (63, 64). While strong HLA-peptide binders are usually found both in experimental and *in silico* data, there is also a population of HLA-II peptides that are either not predicted to bind to a specific allele or that are not detected in the MAPPs assay (56). It is unclear at this stage of discussion whether peptides found uniquely in the MAPPs assay are either false positive (they were non-specifically bound to either an HLA class II receptor or to a plastic surface, for example) or whether HLA class II algorithms did not consider the binding of an HLA class II peptide to its cognate receptor. Conversely, HLA class II peptides only predicted *in silico* might be false negative in the MAPPs assay if the peptide was not amenable to MS detection (65). Nevertheless, *in silico* algorithms do not consider whether the moDC’s processing machinery would generate the expected peptide, or whether the peptide would become truncated or post-translationally modified, which could hinder or enhance the binding of the peptide on the HLA class II receptor. Ideally, some of these questions might be answered using multiple replicates and varying loading amounts of compounds per donor, which is often impractical. Rather, confidence in the data is obtained by identifying multiple peptide copies, charge variants, or length variants within the same sequence regions, therefore avoiding the overinterpretation of outliers. Therefore, researchers should characterize assay variation through dedicated experiments with multiple physical replicates, assessing average variation from cell stimulation to peptide identification via MS.

Recent advances in structure prediction and machine learning may pave the way for novel in silico models that integrate MAPPs assay peptide data with HLA class II structural information. A major hurdle in developing such tools is the limited availability of public data, as much of it remains proprietary to pharmaceutical companies and service providers. While current and emerging in silico approaches still do not accurately predict antigen presentation, they can complement MAPPs assays in applications like de-immunization and hold promise for enhancing future data analysis.

The scope of the MAPPs assay is to identify protein-derived sequences bound to HLA molecules that may potentially be recognized by T cells. The assay does not assess the capacity of these sequences to stimulate T cells, or the nature of these activated T cells. It is therefore critical to consider that not all presented sequences are necessarily deleterious T cell epitopes. Ideally, peptides identified through MAPPs should be further evaluated using a T cell activation assay. Various types of T cell assays are commonly employed, and their harmonization is currently being addressed in a manuscript that will be published by the EIP. If the recognition of the HLA class II peptide candidates cannot be evaluated using a T cell activation assay, for instance, one could envisage a “worst-case” scenario approach by which all presented sequences may be considered as immunogenicity liabilities and considered for de-risking. Of note, if an *in vitro* T cell assay is available, the assay should be qualified so that its false negative rate can be taken into consideration in the overall data interpretation.

In some instances, sequences which were expected to be presented based on their measured or predicted HLA binding affinities are not found in the MAPPs assay. This could be due to the peptide not being generated during protein processing, an overestimation of the binding affinity in the first place (true negatives) or an experimental issue (false negatives). As those cannot be differentiated, one could take a similar conservative approach and mark these sequences low risk rather than no risk.

### 4.10 Recommendations to improve signals and assay robustness

High cell quality of moDCs with a fully differentiated phenotype and high viability are essential for reliable MAPPs assay results. To enhance assay sensitivity, researchers can employ a variety of strategies. For instance, initiating the process with higher numbers of cells, within limits of the analytical instrumentation, can yield a greater number of HLA complexes, thereby increasing the overall concentration of peptides that can be extracted. Additionally, using higher concentrations of the drug will result in increased uptake and processing by moDCs resulting in a higher number of presented peptides derived from the test article. Technically, improving the peptide isolation process by reducing processing steps and surface interactions (for example, by using miniaturized, semi-automated equipment (55) and using latest generation MS instruments will increase the yield of peptides per sample. Interestingly, while the mass spectrometric (“technical”) part of the assay was often seen as the weaker link, mostly due to lack of sensitivity and lack of reproducibility, researchers have now shifted their attention to cell quality and to assay parameters, in particular, to the reagents used to immunoprecipitate the HLA class II receptor-peptide complexes (56). Ultimately, the deployment of ultra-fast and sensitive MS instruments may enable the detection of peptide species with low signal intensity and yield better spectra for database matching.

## 5 Concluding remarks

The HLA class II MAPPs assay has become a major tool to mitigate unwanted immunogenicity of protein therapeutics at the design phase. A technical limitation of the MAPPs assay remains its relatively low throughput, typically 6–8 molecules and controls can be tested in 12–24 donors within a month, which is very low for application early in the drug design process when many molecules are still considered as potential lead candidates. A critical step of the MAPPs assay has been to generate moDCs from monocytes, which requires a steady supply of large quantities of fresh blood and a rather long differentiation protocol. To date, there has been no cellular substitute for moDCs, for example, in the form of stable, immortalized cell lines with sufficient HLA coverage that could be rapidly generated. Other alternatives have been proposed, such as multiplexing the MAPPs assay by combining several test articles in one sample, provided there is sufficient sequence diversity to differentiate the molecules using mass spectrometry. Also, as mentioned in the previous section, semi-automation of the assay using robotic equipment is also being explored. An often-underestimated challenge of the MAPPs assay is database searching, interpretation, and data management. Newer generation mass spectrometers generate very large amounts of data that require robust computing systems and analysis tools to take advantage of this advanced data stream. In this context, automated data analysis tools (52) might assist in reducing manual manipulation of data for decision making to a minimum.

As mentioned earlier, the number of cells required to run the MAPPs assay currently constitutes an important limiting factor for its application beyond blood-derived (e.g., moDC) or high abundance isolated cells (e.g., B cells). For instance, it currently precludes studies of peptide presentation by scarce APCs such as plasmacytoid DCs (66). Once addressed, the feasibility of running the MAPPs assay on cells issued from biopsies could be investigated. If successful, mechanistic studies to elucidate severe adverse events associated with unwanted T cell responses could be envisaged (67). Improving the sensitivity of the method could also extend the application of the MAPPs assay to the detection of potential epitopes derived from low abundance non-coding RNA (ncRNA)-derived peptides. ncRNA-derived peptides represent an emerging class of peptides that originate from RNA types previously regarded as non-coding, namely long non-coding RNAs and circular RNAs (68). Regulation of immune responses in cancer has been described as one application, opening avenues to new treatment approaches (69). Mechanistic studies using the MAPPs assay could contribute to assessing the potential of these peptides as new therapeutic agents.

Extensively used across industry and academia MAPPs assays have been developed by users independently, leading to substantial protocol differences that impair data comparison and interpretation. Here, we reviewed the multiple parameters which contribute to this variability and established comprehensive best practices to implement an efficient assay workflow, increase assay performance, and harmonize data quality, analysis and interpretation across laboratories. Although not without limitations, such as sensitivity, throughput, and sample requirements, the use of MAPPs assays has become a cornerstone in the effort to mitigate immunogenicity during drug development. Further method development resulting in sensitivity increases, reduction in the number of input cells, and enhanced assay capacity is expected to soon broaden the MAPPs assay’s contribution to the discovery and development of safe and efficacious drugs.

## 6 Box summary

- MAPPs assay relevance: The assay plays a crucial role in nonclinical assessment of immunogenicity risk of biotherapeutic candidates by identifying protein-derived sequences that are naturally processed and presented on HLA molecules that T cells might recognize, supporting the biotherapeutic design and selection.
- Challenges: The assay is labor-intensive, relatively low throughput, and protocols vary across different laboratories, which can impact data comparison and interpretation, highlighting the need for aligned best practices.
- Controls: For accurate assay reporting, it is critical to include quality and functional controls, such as unchallenged samples, internal protein controls, and proteins with known antigen presentation patterns. Sensitivity controls for high and low antigen presentation are recommended to better bracket responses. Rigorous control of LC-MS/MS instrumentation via system suitability standards and low-level peptide identification standards is crucial.
- Strategies to enhance assay sensitivity include higher biotherapeutic concentrations and cell numbers to increase uptake and processing by moDCs and total peptide identifications. Improving the peptide isolation process by reducing purification steps and surface interactions, coupled with advanced, high-sensitivity mass spectrometry instruments, can substantially increase peptide identifications.
- Technical Limitations and Considerations: MAPPs assays identify biotherapeutic-derived peptides bound to HLA molecules, though they don’t assess T-cell activation capacity. Major limitations are sensitivity for low abundant peptides and high cell and drug requirements.
- Future Directions: MAPPs analysis of patient samples could further demonstrate and expand the impact of the assay. This will necessitate improved assay sensitivity and reduced cell requirements. Additionally, exploring the feasibility of applying the assay to tissue biopsies, and investigating the application of the assay to detect epitopes derived from low abundance non-coding RNA (ncRNA)-derived peptides could broaden the utility of the MAPPs assay in new therapeutic areas.
